# Development and application of a triplex real-time PCR assay for simultaneous detection of avian influenza virus, Newcastle disease virus, and duck Tembusu virus

**DOI:** 10.1186/s12917-020-02399-z

**Published:** 2020-06-19

**Authors:** Xiyu Zhang, Ming Yao, Zhihui Tang, Daning Xu, Yan Luo, Yunfei Gao, Liping Yan

**Affiliations:** 1 College of Veterinary Medicine, Nanjing Agricultural University, Nanjing, 210095 China; 2 MOE Joint International Research Laboratory of Animal Health and Food Safety, Nanjing Agricultural University, Nanjing, 210095 China; 3 Guangdong Province Key Laboratory of Waterfowl Healthy Breeding, Zhongkai University of Agricultural and Engineering, Guangzhou, 510225 China; 4 Administration for Market Regulation of Guangdong Province Key Laboratory of Supervision for Edible Agricultural Products, Shenzhen Centre of Inspection and Testing for Agricultural Products, Shenzhen, 518000 China; 5 Jofunhwa Biotechnology (Nanjing) Co., Ltd, Nanjing, 211102 China

**Keywords:** Real-time PCR, AIV, NDV, DTMUV, Ducks, Clinical detection

## Abstract

**Background:**

Pathogens including duck-origin avian influenza virus (AIV), duck-origin Newcastle disease virus (NDV) and duck Tembusu virus (DTMUV) posed great harm to ducks and caused great economic losses to the duck industry. In this study, we aim to develop a triplex real-time polymerase chain reaction (PCR) assay to detect these three viruses as early as possible in the suspicious duck flocks.

**Results:**

The detection limit of the triplex real-time PCR for AIV, NDV, and DTMUV was 1 × 10^1^ copies/μL, which was at least 10 times higher than the conventional PCR. In addition, the triplex assay was highly specific, and won’t cross-react with other duck pathogens. Besides, the intra-day relative standard deviation and inter-day relative standard deviation were lower than 4.44% for these viruses at three different concentrations. Finally, a total of 120 clinical samples were evaluated by the triplex real-time PCR, the conventional PCR and virus isolation, and the positive rates for these three methods were 20.83, 21.67, 19.17%, respectively. Taking virus isolation as the gold standard, the diagnostic specificity and positive predictive value of the three viruses were all above 85%, while the diagnostic sensitivity and negative predictive value of the three viruses were all 100%.

**Conclusion:**

The developed triplex real-time PCR is fast, specific and sensitive, and is feasible and effective for the simultaneous detection of AIV, NDV, and DTMUV in ducks.

## Background

China is the largest duck producer of the world, accounting for more than half of the world’s total duck meat stock. During the breeding of ducks, virus infection is a serious problem and has caused huge economic loss to the waterfowl industry, among which, duck-derived avian influenza virus (AIV), duck-derived Newcastle disease virus (NDV), and duck Tembusu virus (DTMUV) are the most common virus. AIV belongs to influenza A virus of *Orthomyxoviridae* and has many serotypes, most of which have been detected in domestic ducks and wild ducks. The high-pathogenicity subtypes such as H5 and H7 are highly virulent to ducks of various ages and breeds [1–3]. Although the low-pathogenicity subtypes such as H3, H6, H9 and H10 are often detected in duck flocks [4–7], ducks often show asymptomatic infection [8]. However when these viruses spread to other birds through polluted water or excreta, they could cause large scale outbreak. NDV belongs to *Avulavirus* of* Paramyxoviridae*, which is highly pathogenic and considered to be a notifiable disease by the World Organisation for Animal Health. Waterfowls are considered as the potential reservoirs for NDV. Since 1997, NDV infection occurred frequently in ducks all over China, causing devastating economic losses [7, 9]. DTMUV belongs to the genus of *flavivirus* in *Flavivirudae* and mainly affects laying ducks. DTMUV can cause egg-laying reducion, death, and reduced feed intake in the infected ducks. The morbidity can reach 90%, and the mortality rate can vary from 5 to 30% when the ducklings are infected with DTMUV [10]. It is worth noting that mixed infection by these three viruses often occurs clinically, and the clinical symptoms of the sick ducks are similar after infected with these viruses, such as thin stools, reduced egg production, fever, and respiratory symptoms, and neurological symptoms. Therefore, establishment of a laboratory diagnostic method for these three viruses is necessary.

At present, the common methods used for detecting these viral infections include virus isolation and identification, serological detection, immuno-electron microscopy, enzyme-linked immunosorbent assay (ELISA) and polymerase chain reaction (PCR) techniques [11–15]. Virus isolation and identification is a confirmation method for AIV, NDV and DTMUV detection [16–19]. However, it requires special facilities, skilled personnel and fresh specimens with viable viruses. Besides, these methods are time-consuming and labor insensitive [20, 21]. Immunoassay-based methods, such as ELISA, have been widely used [22]. However, the difficulty of developing highly specific antibodies and the high false-positive rate limit their application [23]. Although immuno-electron microscopy is an accurate and confirmative method for virus detection [24–26], it is not suitable for clinical diagnosis due to the requirement of complex instruments and a large number of viruses [20].

Nowadays, PCR technology has been accepted as a new gold standard for molecular diagnosis of different pathogens [16, 27–29] because of its high speed, specificity, and sensitivity. So far, conventional PCR methods have been developed for these viruses [30–32]. However, after the amplification of the target fragment, agarose gel electrophoresis was required to analyze the results, which potentially increases the probability of false-positive and is not suitable for clinical rapid detection. As the second-generation PCR technology emerging in 1990s, real-time PCR combines PCR amplification and electrophoresis determination into one step, which saves time and manpower, and reduces the risk of residual contamination. In this study, a TaqMan triplex real-time PCR method was developed and optimized, after validation, it was applied to the real sample analysis. The results showed that developed method is rapid, specific and sensitive, which is feasible and effective for simultaneous detection of AIV, NDV, and DTMUV in ducks.

## Result

### Optimization of the triplex real-time PCR assay

**Fig. 1:**
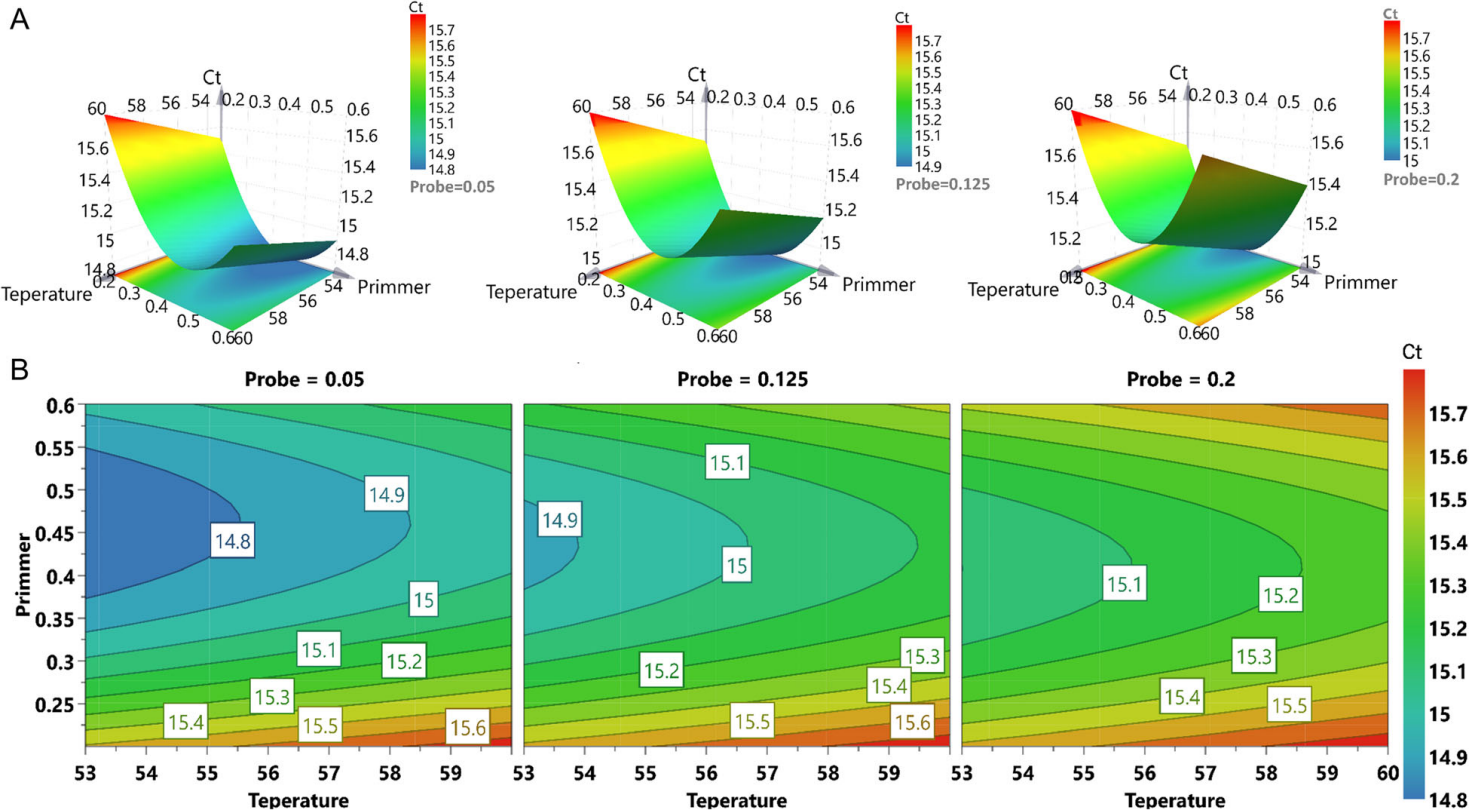
**Response surface plots for AIV.** A: Different combinations of three factors to form the three-dimension response surface curves. B: 4D plots indicated the interaction between the three factors

**Fig. 2:**
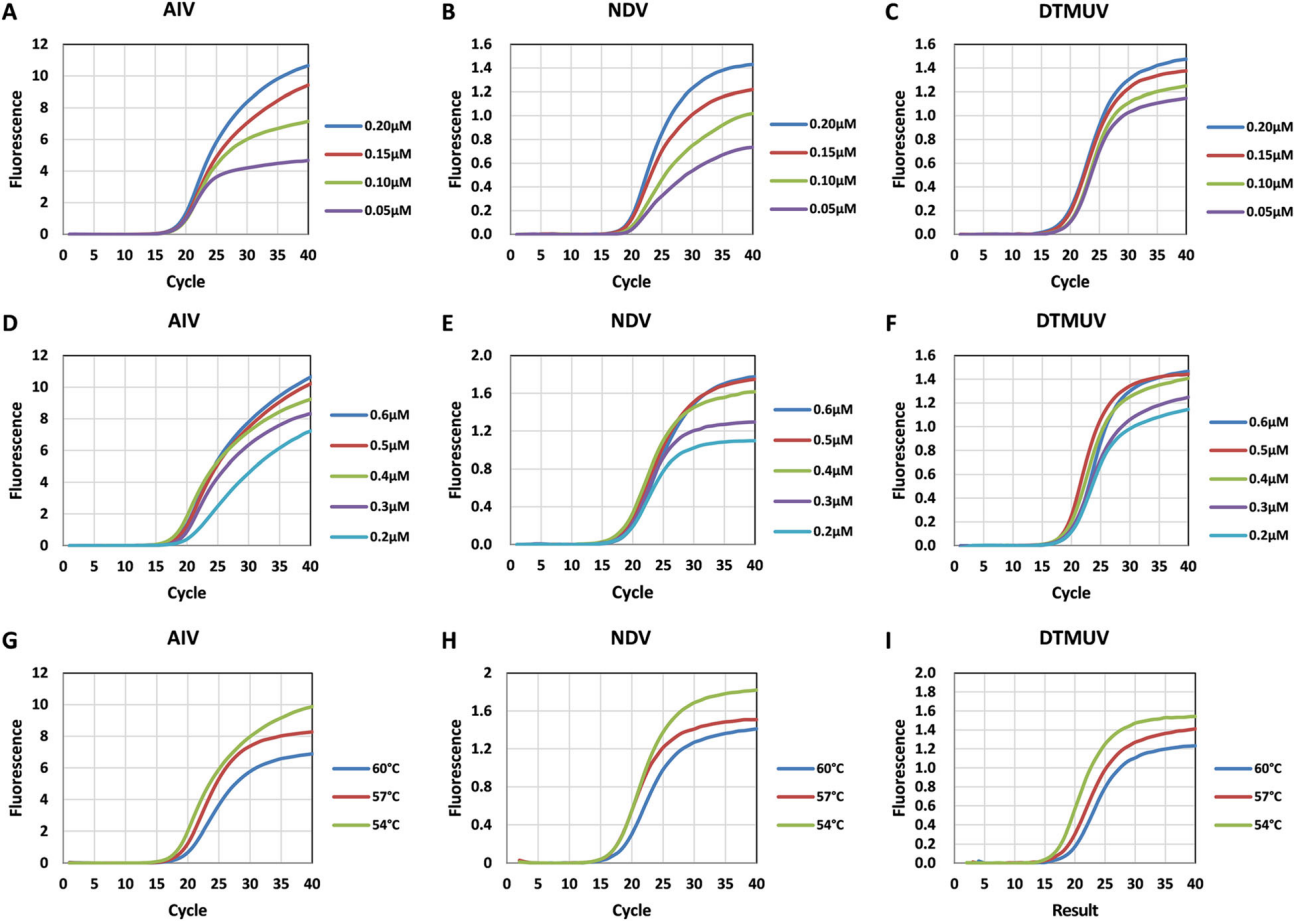
**Optimization of fluorescence intensity.** The effect of different probe concentration on fluorescence signal of AIV (A), NDV (B), DTMUV (C). The effect of different primer concentration on fluorescence signal of AIV (D), NDV (E), DTMUV (F). The effect of different annealing temperature on fluorescence signal of AIV (G), NDV (H), DTMUV (I)

The triplex real-time PCR method was first optimized by D-optimal design and 16 runs were performed in a randomized batch (in triplicate measurements). Taking AIV as an example, the three-dimension response surface curves are shown in Fig. 1A. The 4D plots further illustrated the interaction between the three factors (Fig. 1B). The results show that low Ct values can be obtained at the annealing temperature of 53–55.5 °C, the probe concentration of 0.05–0.125 μM, and the primer concentration of 0.38–0.55 μM. Figure 2 showed the effect of different annealing temperature as well as the concentration of probes and primers on fluorescence signal. As shown in the figure, the fluorescence signal increased with the increase of probe concentration (Fig. 2A, B, C) and the decrease of annealing temperature (Fig. 2G, H, I). When the primer concentration was in the range of 0.4–0.6 μM, the fluorescence intensity was higher and the difference was not significant (Fig. 2D, E, F). Considering the economic and practical factors, the parameters were selected as follows: annealing temperature at 54 °C, primer concentration at 0.5 μM and probe concentration at 0.1 μM. The amplification curve of these three viruses with optimal parameters was shown in Additional file 1.

### Specificity test

**Fig. 3:**
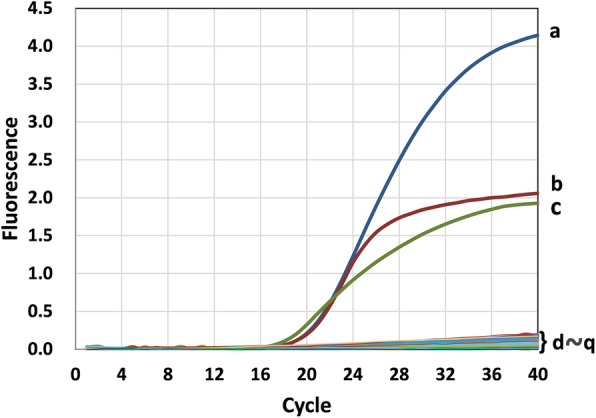
**Specificity of the triplex real-time PCR assay.** The assay was used to amplify AIV, NDV, DTMUV and other pathogens. Only a. pMD-AIV, b. pMD-NDV and c. pMD-DTMUV templates produced positive signals. d. DHAV, e. DEV, f. GPV, g. FAdV, h. EDSV, i. APMV-4, j. APMV-6, k. APMV-8, l. APMV-9, m. *E.coli,* n. SE, o. RA, p. *P. multocida, q. C.perfringens*

The triplex real-time PCR assay was used to amplify other common duck pathogens, including Avian paramyxovirus 4 (APMV-4), Avian paramyxovirus 6 (APMV-6), Avian paramyxovirus 8 (APMV-8), Avian paramyxovirus 9 (APMV-9), *Clostridium perfringens* (*C.perfringens*)*,* duck hepatitis A virus (DHAV), duck enteritis virus (DEV), goose parvovirus (GPV), fowl adenovirus (FAdV), duck egg drop syndrome virus (EDSV), *Escherichia coli* (*E.coli*), *Pasteurella multocida* (*P. multocida*)*, Riemerella anatipestifer* (RA) and *Salmonella* (SE). As speculated, only strong fluorescence signals were obtained for AIV, NDV, and DTMUV. The signals for other samples and negative controls were below the baseline detection levels (Fig. 3), indicating that the triplex real-time PCR method has good specificity.

### Sensitivity test

**Fig. 4:**
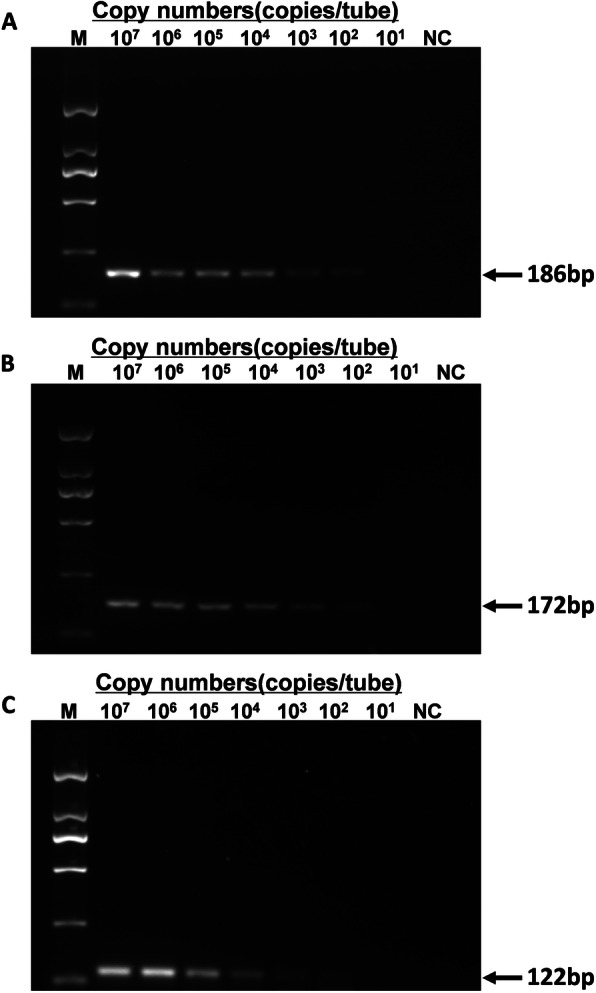
Sensitivity of the conventional PCR method. Templates of pMD-AIV (A), pMD-NDV (B) and pMD-DTMUV (C) were diluted from 1 × 10^7^ copies/μL to 1 × 10^1^ copies/μL. The detection limit of conventional PCR is 1 × 10^2^ copies/μL for AIV, NDV and DTMUV. The gels were cropped. M, DL2000 marker; NC, negative control.

**Fig. 5:**
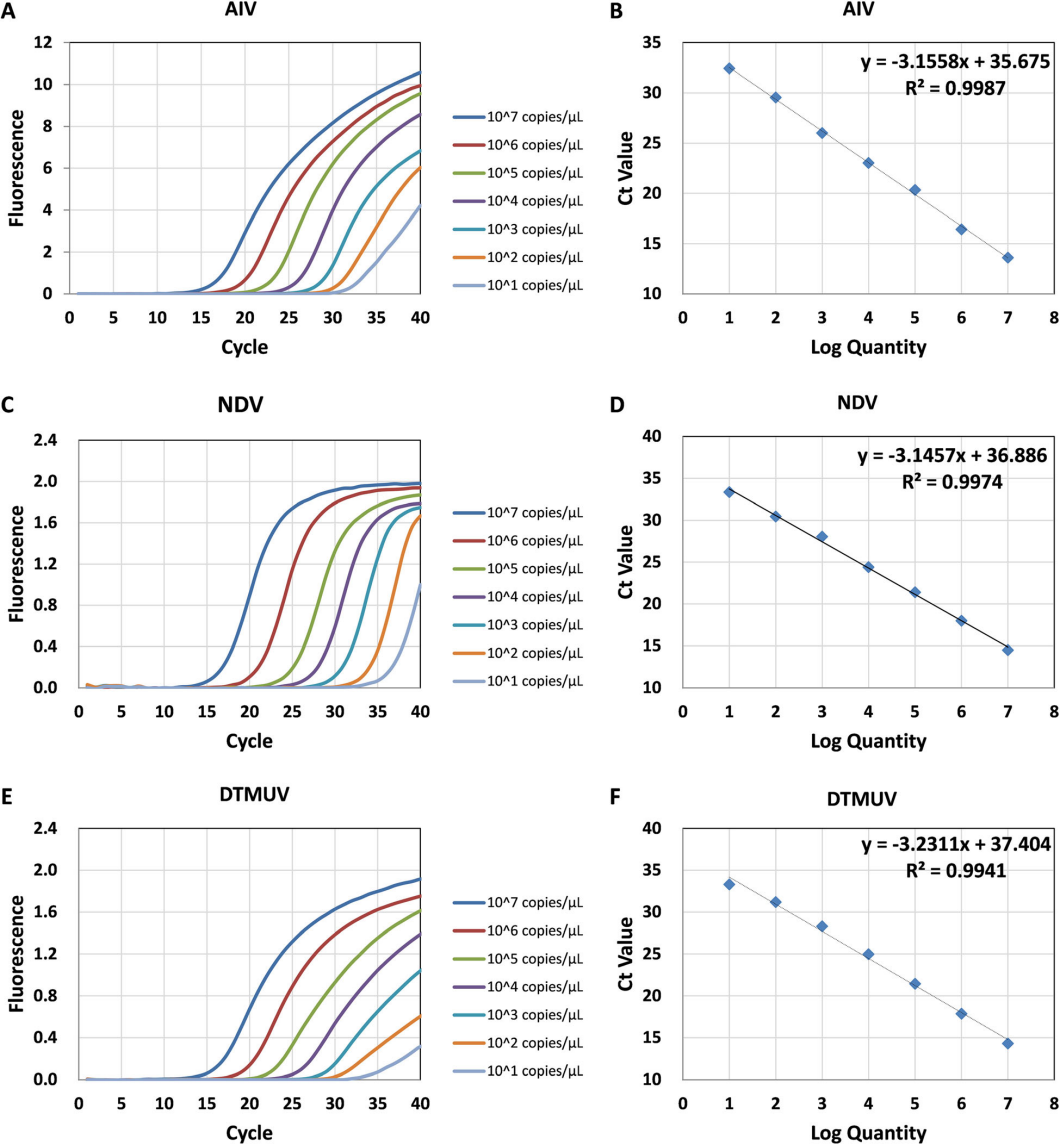
**Standard curves of the triplex real-time PCR assay.** The linear range and detection limit of each virus were evaluated by detecting 10-fold serial dilution of the plasmid (from 1 × 10^7^ copies/μL to 1 × 10^1^ copies/μL) against the Ct. The coefficient of determination (R^2^) and the equation of the regression curve (y) were calculated

Serial dilutions of the standard plasmids (from 1 × 10^7^ copies/μL to 1 × 10^1^ copies/μL) were used to determine the sensitivities of the triplex real-time PCR assay, uniplex real-time PCR (Table 1) and conventional PCR. The standard curve was generated for each virus by 10-fold serial dilutions. As shown in Table 1 and Fig. 4, the detection limits of triplex and uniplex real-time PCR were 1 × 10^1^ copies/μL for these three viruses, while the detection limit for the conventional PCR was 1 × 10^2^ copies/μL for AIV, NDV and DTMUV. That is, the detection limits of real-time PCR were 10 times better than that of the conventional PCR method. The standard curves and amplification curves for the triplex assays were shown in Fig. 5. The SD values were analyzed in triplicate by real-time PCR, which revealed that the results of the assays were reliable and accurate (Table 1).

**Table 1:** Sensitivity and standard curves of the triplex real-time PCR assay

Number of DNA copies (copies/μL)	triplex real-time PCR Ct Value (mean ± SD)			Uniplex real-time PCR Ct Value (mean ± SD)		
	AIV	NDV	DTMUV	AIV	NDV	DTMUV
1 × 10^7^	13.61 ± 0.20	14.50 ± 0.14	14.30 ± 0.33	13.93 ± 0.07	15.01 ± 0.17	14.68 ± 0.41
1 × 10^6^	16.42 ± 0.21	18.00 ± 0.37	17.87 ± 0.09	16.31 ± 0.15	18.22 ± 0.26	18.05 ± 0.04
1 × 10^5^	20.33 ± 0.22	21.39 ± 0.08	21.44 ± 0.09	20.32 ± 0.04	22.26 ± 0.11	21.24 ± 0.16
1 × 10^4^	23.02 ± 0.52	24.41 ± 0.16	24.99 ± 0.05	23.04 ± 0.44	24.71 ± 0.16	24.73 ± 0.43
1 × 10^3^	26.01 ± 0.37	28.04 ± 0.35	28.30 ± 0.08	25.84 ± 0.20	27.74 ± 0.53	27.78 ± 0.01
1 × 10^2^	29.55 ± 0.15	30.45 ± 0.36	31.19 ± 0.65	29.30 ± 0.30	30.78 ± 0.47	31.12 ± 0.12
1 × 10^1^	32.42 ± 0.68	33.34 ± 0.71	33.29 ± 0.86	32.98 ± 0.34	33.85 ± 0.68	33.38 ± 0.60

Serial 10-fold dilutions from 1 × 10^7^ to 1 × 10^1^ copies of pMD-AIV, pMD-NDV, pMD-DTMUV were used as templates to determine the standard curve of the triplex real-time PCR and for uniplex real-time PCR. Each dilution was performed in triplicate for the assays

Ct ≤ 34 was considered positive for AIV, Ct ≤ 35 was considered positive for NDV and DTMUV

### Repeatability and reproducibility of the real-time PCR

The intra- and inter-assay CVs were evaluated at the conventration of 1 × 10^7^, 1 × 10^5^ and 1 × 10^3^ copies/μL, respectively. The results showed that the intra-assay CV were below 2.19%, while that of inter-assay CV were below 4.44% (Table 2). Therefore, the triplex real-time PCR assay developed in this study is highly reliable and accurate.

**Table 2:** Intra- and Inter- assay reproducibility of the triplex real-time PCR

Name	Number of DNA Copies (copies/μL)	Intra-assay			Inter-assay		
		Mean	SD	CV (%)	Mean	SD	CV (%)
AIV	1 × 10^7^	13.71	0.27	1.97	13.52	0.60	4.44
	1 × 10^5^	20.54	0.22	1.07	20.22	0.25	1.24
	1 × 10^3^	25.97	0.35	1.35	26.14	0.61	2.33
NDV	1 × 10^7^	14.72	0.07	0.48	14.64	0.27	1.84
	1 × 10^5^	21.44	0.31	1.45	21.50	0.34	1.58
	1 × 10^3^	28.11	0.35	1.25	27.90	0.76	2.72
DTMUV	1 × 10^7^	14.25	0.12	0.84	14.38	0.33	2.29
	1 × 10^5^	21.35	0.35	1.64	21.46	0.36	1.68
	1 × 10^3^	28.25	0.62	2.19	28.33	0.89	3.14

### Co-infection models and clinical sample detection

As shown in Table 3, the method could detect three viruses at the combinations of different concentrations. Furthermore, the Ct value of the co-infection models also satisfied the linear standard, indicating its applicability in virus quantification during the co-infection.

**Table 3:** The detection of the co-infection models by triplex real-time PCR

Co-infection proportion ^**a**^	Number of DNA copies (copies/μL)			Co-infection real-time PCR Ct Value (mean ± SD) ^**b**^		
	AIV	NDV	DTMUV	AIV	NDV	DTMUV
AIV:NDV:DTMUV = 10:1:1	1 × 10^7^	1 × 10^6^	1 × 10^6^	13.66 ± 0.13	18.15 ± 0.27	18.15 ± 0.27
AIV:NDV:DTMUV = 10:5:1	1 × 10^7^	5 × 10^6^	1 × 10^6^	13.50 ± 0.28	17.25 ± 0.27	18.03 ± 0.16
AIV:NDV:DTMUV = 1:5:10	1 × 10^6^	5 × 10^6^	1 × 10^7^	16.58 ± 0.22	17.13 ± 0.15	14.54 ± 0.28
AIV:NDV = 1:1	1 × 10^7^	1 × 10^7^	–	13.28 ± 0.48	14.43 ± 0.33	–
AIV:NDV = 10:1	1 × 10^7^	1 × 10^6^	–	13.57 ± 0.22	18.25 ± 0.23.	–
AIV:NDV = 100:1	1 × 10^7^	1 × 10^5^	–	13.93 ± 0.10	21.73 ± 0.38	–
AIV:DTMUV =1:1	1 × 10^7^	–	1 × 10^7^	13.43 ± 0.32	–	14.38 ± 0.34
AIV:DTMUV =10:1	1 × 10^7^	–	1 × 10^6^	13.57 ± 0.29	–	17.92 ± 0.32
AIV:DTMUV =100:1	1 × 10^7^	–	1 × 10^5^	13.24 ± 0.34	–	21.33 ± 0.18
NDV:DTMUV =1:1	–	1 × 10^7^	1 × 10^7^	–	14.32 ± 0.32	14.53 ± 0.22
NDV:DTMUV =10:1	–	1 × 10^7^	1 × 10^6^	–	14.49 ± 0.19	18.02 ± 0.14
NDV:DTMUV =100:1	–	1 × 10^7^	1 × 10^5^	–	14.54 ± 0.15	21.68 ± 0.23

^a^ There were two kinds of co-infection models included:the triplex co-infection and duplex co-infection

^b^ The data was shown as the mean ± SD (*n* = 3)

-: represents no design in the corresponding system

Finally, a total of 120 samples were examined (Table 4). As shown in the result, 25 samples (17 of AIV, 7 of NDV, 3 of DTMUV) were determined positive by the triplex real-time PCR method, while 26 samples (18 of AIV, 7 of NDV, 3 of DTMUV) were positive determined by conventional PCR method. However, only 23 samples could be detected by virus isolation (16 of AIV, 6 of NDV, 3 of DTMUV). The virus positive rate were 20.83% (25/120), 21.67% (26/120) and 19.17% (23/120) as detected by triplex Real-time PCR, conventional PCR, and virus isolation. All positive results were verified by sequencing. The 23 positive samples determined by the virus isolation were confirmed by the real-time PCR. However, sample No.17 and No.27, which were determined as AIV positive and NDV positive respectively by real-time PCR, were negative by virus isolation. Furthermore, 2 samples, which were co-infected with AIV and NDV, could be detected by these three methods.

**Table 4:** Results of the clinical positive samples detected by triplex real-time PCR, conventional PCR and virus isolation

Positive samples	AIV			NDV			DTMUV		
	triplex real-time PCR	Conventional PCR	Virus isolation	triplex real-time PCR	Conventional PCR	Virus isolation	triplex real-time PCR	Conventional PCR	Virus isolation
1	+	+	+	–	–	–	–	–	–
2	+	+	+	–	–	–	–	–	–
3	+	+	+	–	–	–	–	–	–
4	+	+	+	–	–	–	–	–	–
5	+	+	+	–	–	–	–	–	–
6	+	+	+	–	–	–	–	–	–
7	+	+	+	–	–	–	–	–	–
8	+	+	+	–	–	–	–	–	–
9	+	+	+	–	–	–	–	–	–
10	+	+	+	–	–	–	–	–	–
11	+	+	+	–	–	–	–	–	–
12	+	+	+	+	+	+	–	–	–
13	+	+	+	+	+	+	–	–	–
14	+	–	+	–	–	–	–	–	–
15	+	–	+	–	–	–	–	–	–
16	+	–	+	–	–	–	–	–	–
17	+	–	–	–	–	–	–	–	–
18	–	+	–	–	–	–	–	–	–
19	–	+	–	–	–	–	–	–	–
20	–	+	–	–	–	–	–	–	–
21	–	+	–	–	–	–	–	–	–
22	–	+	–	–	–	–	–	–	–
23	–	–	–	+	+	+	–	–	–
24	–	–	–	+	+	+	–	–	–
25	–	–	–	+	+	+	–	–	–
26	–	–	–	+	–	+	–	–	–
27	–	–	–	+	+	–	–	–	–
28	–	–	–	–	+	–	–	–	–
29	–	–	–	–	–	–	+	+	+
30	–	–	–	–	–	–	+	+	+
31	–	–	–	–	–	–	+	+	+
Positive rates (%)	14.17 (17/120)	15.00 (18/120)	13.33 (16/120)	5.83 (7/120)	5.83 (7/120)	5.00 (6/120)	7.50 (3/40)	7.50 (3/40)	7.50 (3/40)

The diagnostic performance of real-time PCR and conventional PCR were calculated using the virus isolation as reference (Table 5). In the detection of AIV and NDV, the diagnostic specificity and negative predictive value of the two methods were all above 95%. However, the diagnostic sensitivity and positive predictive value of real-time PCR were better than that of conventional PCR. Furthermore, these two methods have excelent diagnostic performance in the detection of DTMUV, both of which were 100%.

**Table 5:** Clinical performance of triplex real-time PCR and conventianal PCR

Virus	Triplex real-time PCR asssay								
	TP ^**a**^	FN	TN	FP	Tatal	Sensitivity (%)	Specificity(%)	Positive predictive value(%)	Negative predictive value(%)
AIV	16	0	103	1	120	100.00	99.04	94.12	100.00
NDV	6	0	113	1	120	100.00	99.13	85.71	100.00
DTMUV	3	0	37	0	40	100.00	100.00	100.00	100.00
**Virus**	**Conventional PCR methods**								
	**TP**^**a**^	**FN**	**TN**	**FP**	**Tatal**	**Sensitivity(%)**	**Specificity(%)**	**Positive predictive value(%)**	**Negative predictive value(%)**
AIV	13	3	99	5	120	81.25	95.19	72.22	97.06
NDV	5	1	112	2	120	83.33	98.25	71.43	99.12
DTMUV	3	0	37	0	40	100.00	100.00	100.00	100.00

^a^ TP: true positive, FN: false negative, TN: true negative, FP: false positive

## Discussion

For its high specificity and sensitivity, real-time PCR has shown great advantages in the rapid detection of the pathogen [28]. However, the multiplex real-time PCR method to simultaneous detection of AIV, NDV and DTMUV viruses in ducks has not been reported. In this study, we aim to develop a triplex real-time PCR assay that can simultaneously detect these viruses in clinical samples.

Primers and probes are particularly important for the development of a novel triplex real-time PCR method, which determines its sensitivity and specificity. Therefore, in order to find the optimal primers and probes, we made an initial comparison of the sequences of the three viruses, especially the epidemic strains in recent years, and design the primers and probes in the conservative region of each virus (Table 6), the specificity of the primers and probes were verified by BLAST in NCBI. In addition, to guarantee the high amplification efficiency of the developed method, D-optimal design was used to optimize the annealing temperature, primer or probe concentration. In this experiment, if a factor was considered to be critical, the level of the factor at the lowest Ct value would be used in the final experimental protocol [33]. When results conflict occurred, the favourable level of the factor was determined according to the number of minimum response parameters obtained and their economic applicability. Finally, with the necessary compromise, we obtained the ultimate optimum parameters.

**Table 6:** Primers and Probes

Name ^a^	Sequence (5′-3′) ^**b**^	Genomic region	Target genes	Amplicon size(bp)
AIV-F	AGGGTTTGTGTTCACGCTC	180–198		186
AIV-R	CCGGTTGAGTAGCTGAGTGC	346–365	M	
AIV-P	**ROX** - CCGTGCCCAGTGAGCGAGGAC - **BHQ1**	210–233		
NDV-F	GACTCAACTCTTGGGCATACA	837–857		172
NDV-R	TGAGGTGTCAAGCTCTTCTAT	988–1008	F	
NDV-P	**FAM** – CAGTCGGGAACCTAAATAATATGCGTGC - **BHQ-1**	872–899		
DTMUV-F	CAGAGACTGGTTTCATGA	642–659		122
DTMUV-R	AAGCCACCACTGATTGTT	746–763	E	
DTMUV-P	**JOE** - TTACCATGGACAGGGTCATCAGC - **BHQ-2**	667–689		

^a^ F: forward primer, R: reverse primer, P: TaqMan probe

^b^ ROX: 6-carboxy-x-rhodamine, FAM: 6-carboxy-fluorescein, BHQ-1:Black Hole Quencher 1, BHQ-2: Black Hole Quencher

Other viruses and bacteria that may infect ducks were utilized to evaluate the specificity of the triplex real-time PCR method. The results indicated that the primers and probes used in the assay produced neither cross-reactions amongst the three viruses nor nonspecific reactions with other common duck pathogens when all the DNA templates existed in the sample pool. It is worth noting that our AIV primers and probes are designed on the M gene, so a variety of AIV subtypes can be detected. We used the triplex real-time PCR method to amplify H1-H11 AIV subtypes, and the results show that this method is suitable for the detection of these subtypes (Additional file 2).

As shown in the Fig. 5, the coefficient of determination (R^2^) was more than 0.99 in the range of 1 × 10^7^ copies/μL to 1 × 10^1^ copies/μL, thereby indicating the quantitative range of this method. The Ct limit value was defined as the lowest copy number that gives a detectable PCR amplification product at least 95% of the time. When the concentration of AIV was diluted beyond 10 copies/μL, the correlation with the Ct value disappeared, the SD value of the experiment increased, and the Ct value fluctuated randomly between 34 and 36. Based on these results, we chose a Ct value of 34 as the limit value for AIV, corresponding to a detection limit of 1 × 10^1^ copies/μL. Similarly, the Ct limit value of NDV and DTMUV is 35.

In fact, mixed infections of different pathogens at different concentrations are common in clinical practice [34]. When ducks are infected with one virus, the reduced immunity of the body makes them susceptible to other viruses [35], which also results in more serious economic losses [20]. Therefore, the co-infection models was used to determine the detection efficiency of mixed infection. The result demonstrated that the three viruses, at any combination of viruses’ concentration, could be determined, indicating that the triplex real-time PCR was accurate and applicable for clinical sample analysis.

In our previous study, we established uniplex and multiplex conventional PCR methods for the simultaneous detection of 7 viruses [36]. The detection limit of uniplex PCR for AIV, NDV and DTMUV is 1 × 10^1^ copies/μL, 1 × 10^1^ copies/μL, 1 × 10^2^ copies/μL, respectively. However, the sensitivity of the multiplex PCR was not satisfactory, with a detection limit of 1 × 10^4^ copies/μL for these viruses. In addition, many PCR methods were established to detect these viruses [37, 38], with detection limits between 1 × 10^2^ copies/μL and 1 × 10^3^ copies/μL for different viruses. Although these conventional PCR methods are cheap, they are less sensitive and require laborious post-PCR processing, which hinder its application in the sample analysis. Different from the conventional PCR, the real-time PCR method can monitor the amplification of the target in real time, with high sensitivity and fast speed. At present, a variety of uniplex real-time PCR methods have been established for the detection of these viruses, which can detect the minimum 2 × 10^1^ copies/μL of NDV [39] and 4 × 10^2^ copies/μL of DTMUV [40]. Some multiplex real-time PCR methods can distinguish various subtypes of AIV, with detection limits of 1 × 10^1^ copies/μL [41] or 1 × 10^3^ copies/μL [42]. Compared with the above methods, the real-time PCR method established in this experiment realizes the simultaneous detection of AIV, NDV and DTMUV, with a detection limit of 1 × 10^1^ copies/μL, which is suitable for rapid detection of these viruses.

The developed method has been applied for a pilot study of clinical sample and all these viruses were found. It is worth noting that the analysis of the clinical sample indicates that the AIV positive infection rate in the Chinese live poultry market is high. This finding is consistent with the previous report, which showed that the AIV virus has been detected in live poultry market from time to time since the emergence of human infectious AIV in China in 2013 [43]. This situation highlights the need for virus surveillance. To this end, the method developed in the study has great practical value.

## Conclusion

The multiplex real-time PCR described here provides a novel tool for routine diagnosis and epidemiological surveillance of three viruses, AIV, NDV, and DTMUV by allowing rapid detection and quantification of these viruses in the clinical field.

## Methods

### Pathogens and clinical samples

For specificity testing, the nucleic acids of AIV(H1-H11), NDV, DTMUV, APMV-4, APMV-6, APMV-8, APMV-9, EDSV, DHAV, DEV, GPV, FAdV, *E. coli*, SE, RA, *Pasteurella multocida* and *Clostridium perfringens* were used in the study. More details of these pathogens are in the Additional file 3.

With the permission of animal owners and relevant managers, we collected 40 samples (20 tissue specimens and 20 throat swabs) at the Nanjing Tianyin Mountain Agricultural Trade Market on October 17, 2018 and 80 samples (40 tissue specimens and 40 throat swabs) at the Jiangsu Yancheng Wetland National Nature Reserve on November 15, 2018. At the same time, fifty negative samples (25 tissue specimens and 25 throat swabs) were collected from these two locations, which were confirmed to be free of AIV, NDV and DTMUV on real-time PCR [44–46]. All the samples were transported at − 40 °C and kept at − 80 °C for long-term storage. The homogenized tissue specimens and throat swabs were centrifuged at 6000×g for 5 min at 4 °C.

An aliquot of the supernatant of all samples was used to extract proviral RNA, and then reverse transceibed into cDNA, which was utilized as a template for triplex real-time PCR and conventional PCR detection [36]. The remaining supernatant was passed through 0.45-m filters to carry out virus isolation. All viruses were propagated in 9- to 11-day-old specific pathogen-free (SPF) embryonated chicken eggs and harvested from amnioallantoic fluids or tissues of inoculated eggs.

### RNA extraction and cDNA synthesis

The nucleic acid of AIV, NDV, and DTMUV was extracted by Viral RNA Extraction Kit (Sangon Biotech, China), and then reverse transcribed into cDNA by Reverse Transcription Kit (Thermo Scientific, USA), according to the manufacturer’s instructions. Finally, the concentration and purity of each genome were determined by spectrophotometry (Thermo Scientific, USA) and preserved at − 20 °C.

### Primer and probe design

The complete gene sequences of AIV, NDV and DTMUV strains were downloaded from GenBank, and the viral gene sequences were aligned using DNAMAN (LynnonBiosoft, USA) to find gene conserved regions. The target genes including Matrix (M) gene for AIV, Fusion (F) gene for NDV and Envelope (E) gene for DTMUV were highly conserved (Table 6). We designed three pairs of specific primers and probes for each virus using Primer Premier 5 (Premier, Canada) in accordance with the results of sequence alignment. The fluorescent reporter dyes FAM, JOE and ROX were labeled at the 5′ end of the three viruses, and BHQ1, BHQ1, and BHQ2 were linked at the 3′ end for the simultaneous detection of the three genes in a single reaction (Table 6). The primers were synthesized by Nanjing Kingsley Biotechnology Co. Ltd.

### Standard plasmid preparation

Specific target fragments were amplified with the primers (Table 6), and then cloned into the pMD-18 T vector (TaKaRa, China) to obtain the recombinant plasmids pMD-AIV, pMD-NDV, and pMD-DTMUV. The copy number of the recombinant plasmids was calculated using the following formula: copy number (copies/μL) = NA (copies/mol) × concentration (g/μL)/MW (g/mol), where NA is Avogadro’s number and MW is the base number times 340 [47].

### Experimental design for the real-time PCR method

The real-time PCR method was first optimized using a D-optimal design consisting of 16 experiments. Three factors with three levels each were considered. These factors include annealing temperature (from 53 °C to 60 °C), primer concentration for each target gene (from 0.2 μM to 0.6 μM), and probes for each target gene (from 0.05 μM to 0.2 μM). The Ct value was used in statistical analysis. All analyses were performed using MODDE 12.1 software (Umetrics, Sweden). The relationship between the response Y and the variables X_i_, X_j_ was expressed as Y = β_0_ + β_i_X_i_ + β_j_X_j_ + β_ij_X_i_X_j_ + β_ii_X_i_^2^ + β_jj_X_j_^2^ +. .. ε, where β_s_ were the regression coefficients and ε was the experimental error. The linear coefficients β_i_ and β_j_ were the quantitative effect of the respective variables. The cross coefficient β_ij_ measured the interaction between the variables, and the square terms of β_ii_X_i_^2^ and β_jj_X_j_^2^ described the non-linear effects on the response [21]. Then we explored the effects of different annealing temperature, primer and probe concentration on the fluorescence signal. The conditions with the smallest Ct value and highest fluorescence signal were set as the optimal reaction conditions .

### Real-time PCR method

The real-time PCR was determined in a 20.0 μL reaction system with a LightCycler 96 real-time PCR system (Roche, Switzerland). The ingredients were 10.0 μL of TaKaRa Premix Ex Taq™ (Probe real-time PCR), 0.5 μM of the primers for AIV, NDV and DTMUV genes, 0.1 μM of the probes, and 1.0 μL of template. The PCR program was set as follows: pre-denaturation at 95 °C for 2 min and 40 amplification cycles of 95 °C for 10 s and 54 °C for 30 s. Multiple fluorescent signals were obtained once per cycle upon the completion of the extension step. The standard plasmid pMD-AIV, pMD-NDV and pMD-DTMUV were used as the positive control, ddH_2_O as the negative control. The assay was repeated at least three times within the study.

### Conventional PCR method

Conventional PCR assay was conducted in a 20.0 μL reaction system, which containing 10.0 μL of 2× Taq Master Mix (Vazyme Biotech, USA), 0.5 μM of the primers for AIV, NDV, and DTMUV respectively, 1.0 μL of template and ddH_2_O to a final volume of 20.0 μL. The amplification programme was that, pre-denaturation at 95 °C for 5 min, followed by 40 cycles of sequentially denaturation at 95 °C for 30 s, annealing at 54 °C for 30 s and extension at 72 °C for 20 s, a final extension at 72 °C for 10 min. The PCR products were analysed by 1.5% agarose gel electrophoresis. ddH_2_O was used as negative control.

### Validation of the real-time PCR method

The specificity of the triplex real-time PCR was evaluated by cross-reactivity with other duck viruses and bacteria (including APMV-4, APMV-6, APMV-8, APMV-9, EDSV, DHAV, DEV, GPV, FAdV, *Escherichia coli*, SE, RA, *Pasteurella multocida* and *Clostridium perfringens*). The standard plasmid DNAs were 10-fold serially diluted from 1 × 10^7^ copies/μL to 1 × 10^1^ copies/μL for each virus to determine the detection limit of the triplex real-time PCR method. In addition, standard plasmids in three different concentrations (1 × 10^7^, 1 × 10^5^ and 1 × 10^3^ copies/μL) were used as templates to evaluate the repeatability and reproducibility of the real-time PCR method. The coefficients of variation (CV) for the Ct values of the intra- and inter-assay comparisons were determined by testing three replicates of each concentration in a single round of real-time PCR or by repeating three rounds of real-time PCR.

### Pilot study of the real-time PCR method

To evaluated whether the triplex assay could be used as a diagnostic tool in surveillance programs, the co-infection models were first designed to determine the detection efficiency of the developed method. Then, a total of 120 clinical samples were assayed to evaluated the feasibility of the triplex real-time PCR method. The results were further validated by published conventional PCR as well as virus isolation. Using virus isolation as the gold standard, the sensitivity, specificity, positive predictive value and negative predictive value of the three virus detected by real-time PCR and conventional PCR were calculate [39, 48, 49].

## Supplementary information

**Additional file 1.**

**Additional file 2.**

**Additional file 3.**

## Data Availability

All data generated or analyzed during this study are included in this published article and its Additional files.

## Abbreviations

AIV: avian influenza virus
NDV: Newcastle disease virus
DTMUV: duck Tembusu virus
APMV-4: Avian paramyxovirus 4
APMV-6: Avian paramyxovirus 6
APMV-8: Avian paramyxovirus 8
APMV-9: Avian paramyxovirus 9
DHAV: Duck hepatitis A virus
DEV: Duck enteritis virus
GPV: Goose parvovirus
FAdV: Fowl adenovirus
EDSV: Duck egg drop syndrome virus
*E.coli*: *Escherichia coli*
*C.perfringens*: *Clostridium perfringens*
*P. multocida*: *Pasteurella multocida*
SE: *Salmonella*
RA: *Riemerella anatipestifer*
ELISA: Enzyme-linked immune sorbent assay
PCR: Polymerase Chain Reaction
M: DL2000 marker
NC: negative control
NA: Avogadro’s number
MW: base number times
ddH_2_O: double distilled water
FAO: Food and Agriculture Organization
