# Supplementary material for: Development and application of a triplex real-time PCR assay for simultaneous detection of avian influenza virus, Newcastle disease virus, and duck Tembusu virus
Source: BMC Vet Res. 2020 Jun 19;16:203. doi: 10.1186/s12917-020-02399-z (PMC7304117; doi:10.1186/s12917-020-02399-z)
Supplement: Supplementary file 1 — Additional file 1. [file 12917_2020_2399_MOESM1_ESM.docx]

##

**Additional file 1. The amplification curves of these three viruses with optimal parameters**

Templates of pMD-AIV, pMD-NDV and pMD-DTMUV were detected with 1×10^7^ copies/μL

The X-axis represents the cycles, and the Y-axis represents the fluorescence data.
