# Supplementary material for: Development and application of a triplex real-time PCR assay for simultaneous detection of avian influenza virus, Newcastle disease virus, and duck Tembusu virus
Source: BMC Vet Res. 2020 Jun 19;16:203. doi: 10.1186/s12917-020-02399-z (PMC7304117; doi:10.1186/s12917-020-02399-z)
Supplement: Supplementary file 2 — Additional file 2. [file 12917_2020_2399_MOESM2_ESM.docx]

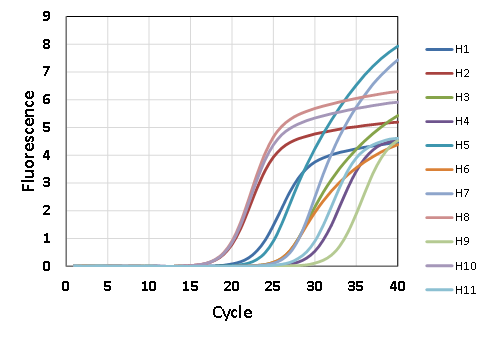


**Additional file 2.** **The amplification curve of AIV subtype H1-H11**

The nucleic acids of AIV subtype H1-H11 were used in the study. And all of them could produce positive signals.
