# Supplementary material for: Development and application of a triplex real-time PCR assay for simultaneous detection of avian influenza virus, Newcastle disease virus, and duck Tembusu virus
Source: BMC Vet Res. 2020 Jun 19;16:203. doi: 10.1186/s12917-020-02399-z (PMC7304117; doi:10.1186/s12917-020-02399-z)
Supplement: Supplementary file 3 — Additional file 3. [file 12917_2020_2399_MOESM3_ESM.docx]

**Additional file 3. The information of duck pathogens**

| **Name** | **Abbreviation** | **Serotype** | **Source of pathogen** |
| --- | --- | --- | --- |
| AIV-H1N1 P2009 | H1 | H1N1 | Maintained in our laboratory |
| AIV-H2N2 21103 | H2 | H2N2 | Maintained in our laboratory |
| AIV-H3N8 11102 | H3 | H3N8 | Maintained in our laboratory |
| AIV- H4N6 20411 | H4 | H4N6 | Maintained in our laboratory |
| AIV-H5N6 060315 | H5 | H5N6 | Maintained in our laboratory |
| AIV-H6N5 20411 | H6 | H6N5 | Maintained in our laboratory |
| AIV-H7N3 201369 | H7 | H7N3 | Maintained in our laboratory |
| AIV-H8N4 20413 | H8 | H8N4 | Maintained in our laboratory |
| AIV-H9N2 201313 | H9 | H9N2 | Maintained in our laboratory |
| AIV-H10N7 20410 | H10 | H10N7 | Maintained in our laboratory |
| AIV-H11N9 21103 | H11 | H11N9 | Maintained in our laboratory |
| NDV La Sota | NDV | - | Sequence synthesis ^a^ |
| DTMUV WF100 | DTMUV | - | Live attenuated vaccine strain ^b^ |
| DHAV A66 | DHAV | serotype 1 | Live attenuated vaccine strain ^c^ |
| DEV C-KCE | DEV | - | Live attenuated vaccine strain ^d^ |
| GPV | GPV | - | Clinical samples |
| FAdV | FAdV | serotype 4 | Clinical samples |
| EDSV AV127 | EDSV | - | Inactivated vaccines ^e^ |
| Avian paramyxovirus 4 | APMV-4 | - | Maintained in our laboratory |
| APMV6 KNU63/2014 | APMV-6 | - | Sequence synthesis ^a^ |
| APMV-8/pintail/Wakuya/20/78 | APMV-8 | - | Sequence synthesis ^a^ |
| PMV-9/ domestic Duck/New York/22/78 | APMV-9 | - | Sequence synthesis ^a^ |
| *Escherichia coli* DE14 | *E.coli*, | serotype O2 | Maintained in our laboratory |
| *Riemerella anatipestifer* P2123 | SA | serotype 6 | Maintained in our laboratory |
| *Clostridium perfringens* NCTC64609 | *C.perfringens* | type A | Maintained in our laboratory |
| *Pasteurella multocida* CVCC274 | *P. multocida* | serotype ST129 | Maintained in our laboratory |
| *Salmonella* CVCC1805 | SE | Serotype *pullorum* | Maintained in our laboratory |

^a^ Sequence synthesis by Sangon Biotech (Shanghai, China)

^b^ purchase from QiLu Animal Health Products Co., Ltd.; Cat. no. 1502522

^c^ purchase from Chengdu Tecbond Biological Products Co., Ltd.; Cat. no. 220012214

^d^ purchase from Guangxi Liyuan Biological Co., Ltd.; Cat. no. 200352023

^e^ purchase from Harbin Pharmaceutical Group Bio-vaccine Co. Ltd; Cat. no.202001

- : represents the virus has only one serotype
